# A Dual-Segmentation Framework for the Automatic Detection and Size Estimation of Shrimp

**DOI:** 10.3390/s25185830

**Published:** 2025-09-18

**Authors:** Malik Muhammad Waqar, Hassan Ali, Heng Zhou, Heba G. Mohamed, Sang Cheol Kim, Michal Strzelecki

**Affiliations:** 1 Division of Electronics and Information Engineering, Jeonbuk National University, Jeonju 54896, Republic of Korea; 202350496@jbnu.ac.kr (H.A.); hengz@jbnu.ac.kr (H.Z.); 2 Core Research Institute of Intelligent Robots, Jeonbuk National University, Jeonju 54896, Republic of Korea; sckim7777@jbnu.ac.kr; 3 Department of Electrical Engineering, College of Engineering, Princess Nourah Bint Abdulrahman University, P.O. Box 84428, Riyadh 11671, Saudi Arabia; hegmohamed@pnu.edu.sa; 4 Institute of Electronics, Lodz University of Technology, 93-590 Lodz, Poland; michal.strzelecki@p.lodz.pl

**Keywords:** computer vision, deep learning, instance segmentation, semantic segmentation, shrimp centerline extraction, aquaculture

## Abstract

In shrimp farming, determining the physical traits of shrimp is vital for assessing their health and growth. One of the critical traits is their size, as it serves as a key indicator of growth rates, biomass, and effective feed management. However, the accurate measurement of shrimp size is challenged by factors such as their naturally curved body posture, frequent overlapping among individuals, and their tendency to blend with the background, all of which hinder precise size estimation. Traditional methods for measuring the size of shrimp involve manual sampling, which is labor-intensive and time consuming. In contrast, image processing and classical computer vision techniques provide some reasonable results but often suffer from inaccuracies, making them unsuitable for large-scale monitoring. To address this problem, this paper proposes a dual-segmentation deep learning-based framework for accurately estimating shrimp size. It integrates instance segmentation using the RTMDet-m model with an enhanced semantic segmentation model to effectively predict the centerline of the shrimp’s body, enabling precise size measurements. The first stage employs the RTMDet-m model for the instance segmentation of shrimp, achieving an average precision (${\mathrm{AP}}_{50}$) of 96% with fewer parameters and the highest frames per second (FPS) count among state-of-the-art models. The second stage utilizes our custom segmentation model for centerline predictive module, attaining the highest FPS and F1-score of 88.3%. The proposed framework achieves the lowest mean absolute error of 1.02 cm and a root mean square error of 1.27 cm in shrimp size estimation compared to the baseline methods discussed in comparative study sections. Our proposed dual-segmentation framework outperforms both traditional and deep learning based methods used for measuring shrimp size.

## 1. Introduction

Shrimp farming plays an important role in global seafood production, and farmed shrimp represents 63% of total shrimp production in 2022 [1]. The marketplace is expanding due to the increase in the number of shrimp-based products. In order to increase shrimp production, careful feeding strategies, environment management, and monitoring their health [2,3,4] are required. To gain insights into these factors, it is important to measure the physical traits of the shrimp. In shrimp farming, accurately estimating shrimp size is vital for assessing overall health, monitoring growth patterns, grading, and estimating biomass [4,5]. Measuring the size of shrimp is crucial for feed optimization, making harvesting decisions, and ensuring sustainable farm management. A significant amount of research has focused on size estimation in aquaculture; however, most studies have focused on fish rather than shrimp. Additionally, the proposed methods are influenced by the fish’s pose and orientation, and they require a specialized control environment for accurate size measurement. The following paragraph provides a detailed discussion of existing methods employed for size estimation in both fish and shrimp along with their limitations.

The automated size measurement methods are crucial for managing aquaculture species, as they offer a more efficient and accurate alternative to traditional manual methods [6]. The recent advancements in computer vision and image processing have greatly improved automation in aquaculture by tackling various challenges related to counting, detection, segmentation, and size measurement [7,8,9,10,11]. In earlier research, Hsieh et al. [12] proposed an image-based method for estimating the snout-to-fork length (SNFL) of tuna on fishing vessels using handheld cameras and Hough transform techniques. This method reduced manual handling but required operator training for proper camera positioning and was sensitive to the angle of image capture. Miranda et al. [13] developed a polynomial curve-fitting method (PCFM) to measure the size of rainbow trout. Fish swim through a channel, and a camera captures images to create a third-order polynomial curve that predicts size. However, this method requires the fish to be in a tube, which is not ideal for aquaculture settings. It also necessitates manual selection of the polynomial degree, which may not generalize well due to variations in fish orientation and posture. These methods are limited in their ability to handle curved body postures and varying shapes, whereas deep learning approaches can robustly capture complex body shapes and features, thereby providing more accurate and reliable measurements.

Tseng et al. [14] developed a patch-wise CNN classifier for identifying fish body parts to estimate size by projecting these regions onto the original image using a pixel-to-distance ratio. The method performed well in controlled settings but struggles with multiple fish and real-time processing due to its computationally intensive sliding-window approach. In a recent study, Wang et al. [3] present a method to automatically measure shrimp size. It uses a U-shaped fully convolutional network along with second-order anisotropic Gaussian kernels. This method relies on silhouette segmentation results of shrimp; however, it is not effective in scenarios with dense shrimp due to the weak segmentation model. Zhao et al. [15] proposed a heat-to-tail matching algorithm for identifying fish heads and tails, which helps with differentiating them and predicting their size. However, distinguishing contours can be difficult in dense clusters, and the assumption that each fish has both head and tail information may not always be valid. Zhou et al. [16] present a binocular stereo vision method to measure fish size by extracting a center line. They use the GrabCut algorithm for segmentation, but its inaccuracies and the deburring process can affect measurement precision. Rodrigo et al. [17] recently proposed a Voronoi diagram-based method (VDBM), which is an automated method for 3D centerline extraction in coronary arteries using the Voronoi diagram. This method is effective in extracting the centerline. However, it requires post-processing to eliminate extraneous branches, which increases overhead and reduces throughput, impacting real-time usability.

To address the limitations of previous studies, this work proposes a dual-segmentation deep learning framework for accurate shrimp size measurement. Unlike traditional approaches, the proposed framework does not require manual handling, specialized settings, or predefined assumptions. It is capable of delivering reliable predictions even in the presence of multiple shrimp instances or overlapping conditions. Furthermore, the method requires only minimal post-processing, which is achieved through the skeletonization algorithm proposed by Zhang et al. [18]. Our framework consists two segmentation models along with a skeletonization operator to achieve impressive results. The first segmentation model employs the RTMDet-m model for instance segmentation, allowing us to isolate individual shrimp. The second model is our custom segmentation model designed to perform precise centerline extraction based on the outputs from the instance segmentation model. Our proposed framework outperforms earlier methods for measuring shrimp size in terms of error metric and processing time. The main contributions of this study are summarized as follows:

1. This study introduces a novel framework that integrates instance and semantic segmentation to efficiently extract the centerline of individual shrimp without requiring any post-processing step. Additionally, our proposed framework is robust to variations in the pose or orientation of the shrimp.
2. An enhancement to the baseline semantic segmentation model was proposed to improve the accuracy of shrimp centerline prediction. Our proposed model outperforms other baseline approaches significantly and prevents the creation of burrs along the centerline.
3. Furthermore, our study presents two comprehensive datasets designed to advance research in shrimp instance segmentation and size estimation. The dataset comprises high-quality images collected from three diverse environments with varying numbers of shrimp.

## 2. Materials and Methods

This section provides a comprehensive overview of the experimental setup, dataset, proposed framework, and its components to estimate shrimp size. Section 2.1 outlines the experimental setup used to conduct the experiments. Section 2.2 describes the two custom datasets utilized in this study. Section 2.3 presents an overall overview of the proposed framework for estimating shrimp size. Section 2.4 details our first model within the framework, specifically the instance segmentation model. Section 2.5 provides information about our custom segmentation model in the centerline predictive module and the skeletonization operator used to derive a one-pixel-wide centerline representation.

### 2.1. Experimental Setup

The experimental setup for estimating shrimp size consists of shrimp, a rectangular container, a camera, and a laptop. The shrimp used to create the dataset, which ranged from 4 cm to 11 cm in length, were collected from a farm and placed in a container. The container has internal dimensions of 50 cm (length) × 35 cm (width) × 25 cm (depth) and was filled with water up to a depth of 3–5 cm. This preserves the natural posture and mobility of the shrimp while keeping them within the imaging setup. The video data were recorded under controlled laboratory conditions using an Intel RealSense Depth Camera D435 (Intel Corporation, Santa Clara, CA, USA), which operated in RGB mode at 30 frames per second (FPS) with a resolution of 848 × 480 pixels. The camera was mounted centrally on the container lid at a fixed vertical distance of 25 cm above the water surface to ensure consistent coverage. To capture variations in visual conditions, three experimental environments were prepared by varying the shrimp density. Subsequently, the recorded video streams were used to extract the image frames from them uniformly. The details on the construction of the dataset from these recordings are provided in the following section.

### 2.2. Dataset

**Figure 1:**
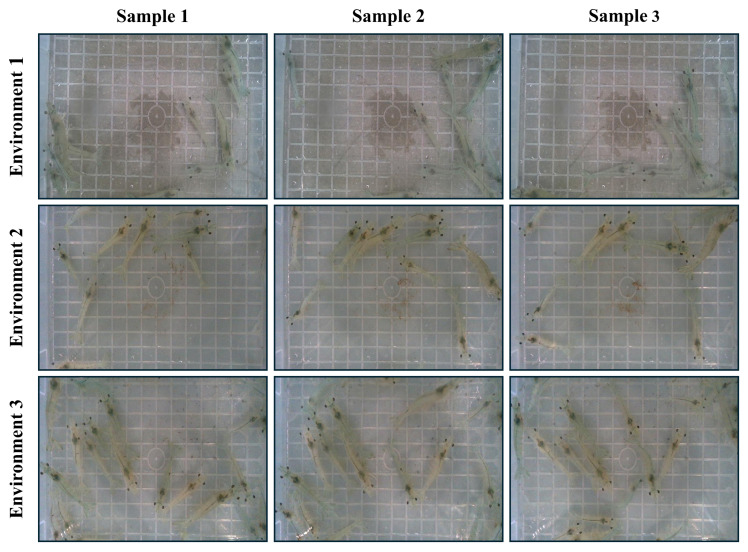
Extracted images for instance segmentation dataset collected from three diverse environments, varying in number of shrimps.

This study employs two specialized shrimp datasets, which were developed to enable the experimentation and validation of our framework for estimating shrimp size in real time. The first dataset is an instance segmentation dataset that comprises 1000 high-quality images collected from 3 different environmental video streams, each with varying shrimp densities. Environments 1, 2, and 3 differed in shrimp counts, with the numbers ranging between 10 and 18. The first environment contained 10 shrimps, the second environment contained 15, and the third environment contained 18. Due to the live movement of shrimps, the dataset comprises diverse images, including nonoccluded and occluded cases. Each image was precisely annotated, and instance segmentation masks were generated for each individual shrimp. The visualization of the instance segmentation dataset from three distinct environments is provided in Figure 1.

**Figure 2:**
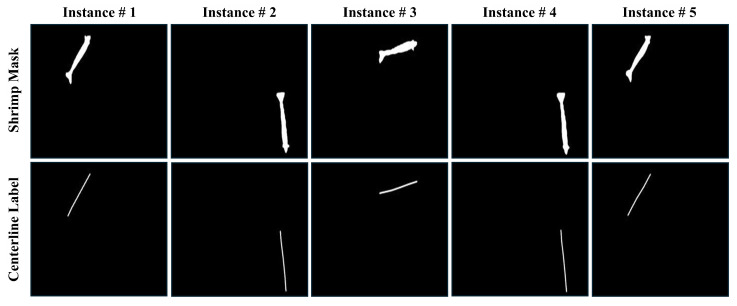
Visualization of centerline dataset for semantic segmentation with shrimp masks in the top row and corresponding centerline labels at the bottom.

The second dataset, known as the mask-to-centerline dataset, was constructed to aid in semantic segmentation for the extraction of the shrimp centerline. To speed up the labeling of the shrimp mask and create the mask-to-centerline dataset, a better strategy was adopted. An instance segmentation model was first trained on a small portion of the dataset with reasonable accuracy. The trained model was then utilized to obtain mask predictions, which were carefully filtered to remove any low-quality masks. This process guarantees high-quality data for training our custom segmentation model for the centerline prediction of shrimp. These data contain approximately 2400 shrimp mask instances, and each mask was manually labeled with a corresponding centerline. Figure 2 provides a visualization of mask samples and their ground truth centerline labels from our second dataset.

The total images in both datasets were divided into three parts: 80% for training, 10% for validation, and 10% for testing. The open-source Computer Vision Annotation Tool (CVAT, version 2.30.0) was used to label the images. Both of our datasets are instrumental in developing models that can accurately segment and estimate the size of shrimp. The following section provides a detailed description of the proposed framework and the models used to develop the shrimp size estimation system.

### 2.3. Proposed Framework Overview

**Figure 3:**
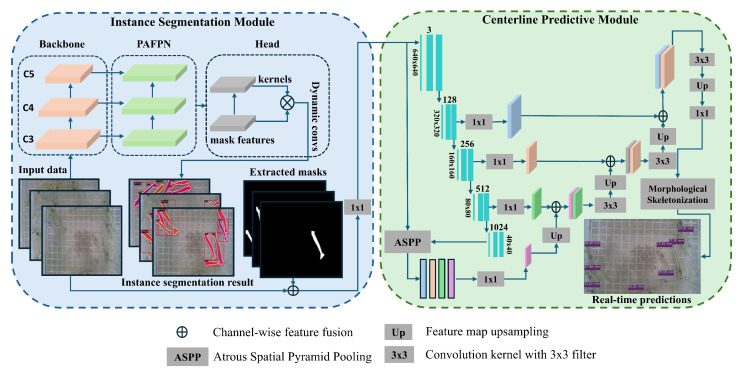
System overview of our proposed dual-segmentation framework for segmentation and size estimation of shrimp.

Our proposed dual-segmentation framework for estimating shrimp size consists of two sequential modules: the instance segmentation module and the centerline predictive module. The instance segmentation module utilizes an instance segmentation model to predict the mask of individual shrimp. In contrast, the centerline prediction module uses our custom segmentation model to predict the centerline of each shrimp. Each of these models is trained independently and operates sequentially during inference. During inference, the original RGB image is passed to the instance segmentation model to isolate individual shrimp precisely. The masks obtained from the instance segmentation are concatenated with the RGB image to form a four-dimensional feature map. This feature map is then reduced to a three-dimensional feature map using a 1 × 1 convolution filter. The resultant feature would serve as input to our second custom segmentation model that provides precise centerline predictions for each shrimp. The extracted centerline from the second module is passed to a morphological operator to obtain a single-pixel line. Our framework is optimized to ensure fast and accurate processing. An overview of the proposed framework is illustrated in Figure 3.

The working principal of the instance segmentation model and our proposed segmentation model is discussed in the following Section 2.4 and Section 2.5, respectively.

### 2.4. Instance Segmentation of Shrimp

The first stage of our dual-segmentation framework, which measures the size of the shrimp, is the instance segmentation stage. This stage is essential for detecting and isolating individual shrimp from images, as it directly affects the precision of the subsequent stage for center line prediction. In this study, the RTMDet [19] model was employed specifically for shrimp instance segmentation. This model is integral to our framework due to its high accuracy and real-time performance capabilities. RTMDet uses a fully convolutional approach, which is different from two-stage instance segmentation models like Mask R-CNN that rely on regions of interest (ROIs) to predict the mask precisely. Its advanced mask prediction module allows instance segmentation to be performed directly from the feature maps, which improves FPS.

#### Model Selection for Instance Segmentation of Shrimp

The real-time segmentation of shrimp poses a significant challenge that demands both high-precision modeling and robust generalization capabilities. The primary difficulties stem from the physical characteristics of shrimps themselves. Shrimps are naturally semi-transparent or translucent, which causes them to blend easily into backgrounds, particularly in aquatic environments or on light-colored surfaces. Additionally, shrimps can assume various postures—curved, twisted, or overlapping with other shrimps—which further complicates the task of distinguishing individual instances. These visual ambiguities can lead to either under-segmentation, where parts of a shrimp are missed, or over-segmentation, where multiple shrimps are merged into one another. To address these challenges, it was crucial to find a model that could accurately detect and segment each shrimp under different visual conditions. A comparative analysis of several advanced instance segmentation algorithms was performed, including SOLOv2 [20], YOLACT [21], CondInst [22], SparseInst [23], YOLOv8-m, and RTMDet-m. Among these options, RTMDet consistently showed superior segmentation quality and computational efficiency performance. During our experiments, RTMDet achieved a competitive FPS while still maintaining a high average precision (AP). In addition to accuracy, the need for real-time performance significantly influenced our choice of model. The RTMDet model used in our framework is based on the RTMDet-ins-m variant, which is specifically designed for instance segmentation tasks. The architecture of the selected RTMDet-ins-M model comprises several essential components, which are detailed as follows.

Given an input image $I\in R^{H\times W\times 3}$, RTMDet predicts a set of instance masks ${M_{i=1}}^{K}$, where $M_{i}\in {\left\{0,1\right\}}^{H\times W}$ denotes the binary mask corresponding to the $i^{\mathrm{th}}$ shrimp instance. The model follows a fully convolutional pipeline comprising three major components: a strong backbone CSPNeXt [24], a BiFusion neck for multi-scale feature aggregation, and a decoupled detection head that independently predicts classification, bounding boxes, and segmentation masks. The input image $I\in R^{H\times W\times 3}$ is first passed through a deep convolutional neural network (e.g., CSPNeXt in RTMDet) to extract multi-scale feature maps:

(1) $$F=B\left(I\right),\;F={\left\{F_{l}\right\}}_{l=1}^{L},\;F_{l}\in R^{H_{l}\times W_{l}\times C_{l}}$$

where B(·) denotes the backbone network, and *l* indexes the feature level in the feature pyramid. The feature maps *F* are passed through the detection head to produce three outputs. First, the classification branch outputs the class logits $P_{\mathrm{cls}}\in {\left[0,1\right]}^{H_{l}\times W_{l}\times C}$, where *C* is the number of classes. Second, the regression branch produces the bounding box coordinates $P_{\mathrm{reg}}\in R^{H_{l}\times W_{l}\times 4}$. Third, for instance segmentation, a mask branch generates mask prediction features $P_{\mathrm{mask}}\in R^{H_{l}\times W_{l}\times D}$, where *D* is the dimensionality of the dynamic mask features. The segmentation head uses dynamic convolutional layers to create pixel-level masks directly from feature maps, enabling dense predictions without requiring ROI alignment. The mask predictions improve through aligned convolution and point-wise segmentation branches that enhance boundary localization. The final generated mask can be defined as below:

(2) $${\hat{M}}^{i}=H_{\mathrm{dyn}}\left(P_{\mathrm{mask}},\theta_{i}\right),\;\theta_{i}=\mathrm{Instance}-\mathrm{Specific}\;\mathrm{Parameters}$$

where $H_{\mathrm{dyn}}$ denotes the dynamic convolution operator conditioned on per-instance features to produce the binary mask ${\hat{M}}^{i}\in {\left[0,1\right]}^{H\times W}$. The loss function of the RTMDet model is composed of three main terms: classification loss ($L_{\mathrm{cls}}$), bounding box regression loss ($L_{\mathrm{reg}}$), and mask loss ($L_{\mathrm{mask}}$), which were typically implemented using Dice loss or Binary Cross-Entropy. The total loss $L_{\mathrm{total}}$ in RTMDet combines three key components: classification loss, regression loss, and mask loss. The classification loss uses the focal loss to handle class imbalance and is defined as $L_{\mathrm{cls}}=\sum_{i=1}^{K}\mathrm{FL}\left(P_{\mathrm{cls}}^{i},y_{\mathrm{cls}}^{i}\right)$, where FL(·) denotes the focal loss between the predicted and ground truth class labels. The regression loss, which measures the quality of the predicted bounding boxes, uses the Generalized IoU (GIoU) and is computed as $L_{\mathrm{reg}}=\sum_{i=1}^{K}\mathrm{GIoU}\left(P_{\mathrm{box}}^{i},y_{\mathrm{box}}^{i}\right)$, where $y_{\mathrm{box}}^{i}\in R^{4}$ are the ground truth box coordinates. The mask loss applies a pixel-wise binary cross-entropy (BCE) between each predicted instance mask and the ground truth mask, which is defined as $L_{\mathrm{mask}}=\sum_{i=1}^{K}\mathrm{BCE}\left({\hat{M}}^{i},M^{i}\right)$. These components are combined into a single loss function:

(3) $$L_{\mathrm{total}}=\sum_{i=1}^{K}\left[\mathrm{FL}\left(P_{\mathrm{cls}}^{i},y_{\mathrm{cls}}^{i}\right)+\alpha \cdot \mathrm{GIoU}\left(P_{\mathrm{box}}^{i},y_{\mathrm{box}}^{i}\right)+\beta \cdot \mathrm{BCE}\left({\hat{M}}^{i},M^{i}\right)\right]$$

where α and β are weighting factors that balance the contributions of the box regression and mask prediction losses.

### 2.5. Shrimp Centerline Prediction via Semantic Segmentation

The second stage of the framework addresses the challenge of predicting shrimp centerlines from the instance masks produced in the first stage. The classical image processing methods, such as morphological skeletonization or medial axis transformation, cannot be directly applied to binary masks to obtain their centerlines. These methods tend to create burrs, particularly around the head and tail sides of the shrimp, making them unsuitable for our application. Furthermore, these methods require substantial post-processing to extract a single clean centerline from these skeletons. Post-processing includes pruning unnecessary branches, resolving junctions, and navigating the skeleton graph using custom heuristics or shortest path algorithms. These steps are computationally intensive, difficult to generalize across different shrimp shapes, and ultimately hinder the feasibility of real-time processing.

**Figure 4:**
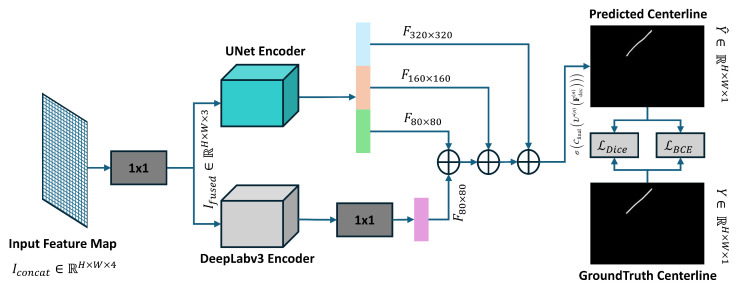
Our proposed segmentation model for the prediction of shrimp centerline with enhanced decoder compared to DeepLabv3 model.

To address these challenges, this study proposed a custom segmentation model, illustrated in Figure 4, to precisely predict the centerline. The segmentation model takes a three-channel image, which consists of a fused RGB image and a binary instance mask, as input and generates a centerline for the shrimp’s body. This method is used to specifically capture the precise structure of the shrimp’s centerline.

#### 2.5.1. Proposed Model for Shrimp Centerline Predictive Module

To address the challenge of accurately extracting the shrimp centerline, this study proposed an enhanced segmentation model built upon the baseline DeepLabV3 model. The proposed segmentation model integrates the outputs of UNet and the DeepLabv3 model to enhance the accuracy of centerline construction. This integration enables better preservation of fine-grained details, which are essential for the prediction of the centerline structure. This novel modification to the DeepLabv3 model achieves more precise centerline predictions. Our proposed segmentation model addresses the following key challenges in centerline segmentation: (1) capturing global contextual cues to resolve spatial ambiguity, and (2) preserving fine spatial details necessary for defining narrow structures. The original DeepLabv3 model utilizes Atrous Spatial Pyramid Pooling (ASPP) to capture multi-scale context, but it does not explicitly incorporate mechanisms for recovering high-resolution details. To address this limitation, the strength of a UNet-like structure is combined with DeepLabv3’s multi-scale contextual learning. This integration leads to a network with enhanced semantic precision and spatial resolution.

To train our proposed segmentation model, an efficient training strategy was adopted, which enhances the model’s generalizability and contextual awareness in centerline prediction. At first, a multi-branch design was employed to merge the original RGB image with the extracted binary mask using a lightweight 1 × 1 convolution operation. This fusion effectively integrates prior information regarding the object’s location, specifically the shrimp, directly into the input of our proposed segmentation model. The workflow of our proposed centerline predictive module is discussed as follows:

The RGB image $I\in R^{H\times W\times 3}$ is concatenated with a binary mask $M_{i}\in {\left\{0,1\right\}}^{H\times W}$ along the channel dimension to produce a four-channel input tensor:

(4) $$I_{\mathrm{concat}}=\mathrm{Concat}\left(I,M_{i}\right)\in R^{H\times W\times 4}$$

This fused input contains both color information and spatial object cues. To reduce the number of channels and retain compatibility with existing architectures, a 1×1 convolution is applied:

(5) $$I_{\mathrm{fused}}={\mathrm{Conv}}_{1\times 1}\left(I_{\mathrm{concat}}\right)\in R^{H\times W\times 3}$$

The resulting $I_{\mathrm{fused}}$ is fed simultaneously into two parallel modules: a U-Net encoder that extracts hierarchical feature representations, and an Atrous Spatial Pyramid Pooling (ASPP) module that captures multi-scale contextual information using parallel atrous convolutions with different dilation rates. The deepest feature map from the encoder is concatenated with a downsampled version of the fused image to guide the ASPP with additional spatial cues. The ASPP output is then upsampled and fused with skip connections from the U-Net encoder. Each fusion stage is followed by convolutional refinement layers. Finally, the decoder produces a centerline mask through a final convolution and sigmoid activation:

(6) $$\hat{Y}=\sigma \left(C_{\mathrm{final}}\left(U^{\left(n\right)}\left(F_{\mathrm{dec}}^{\left(n\right)}\right)\right)\right)\in R^{H\times W\times 1}$$

where $F_{\mathrm{dec}}^{\left(n\right)}$ is a high-resolution, semantically rich feature representation of the image after all encoder–decoder processing. $U^{\left(n\right)}$ denotes the final upsampling operation, $C_{\mathrm{final}}$ represents the final convolutional layer after which raw logits are converted into probabilities using the σ function. The final predicted binary centerline mask is denoted by $\hat{Y}$. This multi-stage process effectively combines hierarchical encoder features, raw RGB cues, and multi-scale context to produce a highly detailed and structurally accurate centerline segmentation output. The architecture of our improved segmentation model for the shrimp centerline prediction is contained in the centerline predictive module in Figure 3. During training, the ground truth centerline is represented as a three-pixel-wide binary mask to improve gradient flow and learning stability. The model is optimized using a composite loss function that combines Dice loss and Binary Cross-Entropy (BCE) loss.

(7) $$L_{\mathrm{seg}}=L_{\mathrm{Dice}}+\beta L_{\mathrm{BCE}}$$

(8) $$L_{\mathrm{seg}}=\left(1-\frac{2\sum_{i}Y_{i}{\hat{Y}}_{i}+\epsilon }{\sum_{i}Y_{i}+\sum_{i}{\hat{Y}}_{i}+\epsilon }\right)+\beta \left(-\frac{1}{N}\sum_{i=1}^{N}\left[Y_{i}\log\left({\hat{Y}}_{i}+\epsilon \right)+\left(1-Y_{i}\right)\log\left(1-{\hat{Y}}_{i}+\epsilon \right)\right]\right)$$

where $L_{\mathrm{Dice}}$ addresses class imbalance and promotes overlap between prediction and ground truth, and $L_{\mathrm{BCE}}$ penalizes pixel-wise misclassification. $Y_{i}$ presents the ground-truth label at pixel *i* and ${\hat{Y}}_{i}$ represents the predicted probability at pixel *i*. The ϵ is there for numerical stability to prevent the zero division, and the scalar β is used to balance the contribution of the two loss terms. In semantic segmentation tasks, especially those involving thin structures like shrimp centerlines, a significant challenge arises due to class imbalance. In these cases, the majority of pixels in the training images belong to the background class, while the foreground (the centerline) occupies only a small portion of the image. This imbalance can result in models being biased toward predicting the background and neglecting the essential foreground structures. The Dice loss function is particularly effective in addressing this issue, as it emphasizes the overlap between the predicted regions and the ground truth. This helps to mitigate the adverse effects of class imbalance and ensures better segmentation of the foreground class.

#### 2.5.2. Centerline Extraction for Size Estimation

After centerline extraction, a skeletonization algorithm was applied to transform the binary map into a one-pixel-wide centerline. This algorithm is based on the method proposed by Zhang and Suen [18] and follows an iterative thinning process. The algorithm removes pixels from the edges of binary shapes while keeping the object’s connections and structure intact. It does this by eliminating boundary pixels in stages, making sure the object stays connected. In each stage, the surrounding 3×3 area of a pixel is represented as an 8-bit value.

(9) $$N\left(p\right)=\sum_{i=0}^{7}2^{i}\cdot b_{i}$$

where $b_{i}\in \left\{0,1\right\}$ represents the binary value of the *i*-th neighbor of pixel *p*. The process continues until no more pixels can be removed, resulting in a clean, one-pixel-wide centerline. The final shrimp’s size is calculated by measuring the arc length of this centerline in pixels. Then, we convert the pixel size to millimeters using a conversion factor obtained from our image setup. The performance of our shrimp size estimation method was evaluated using the Mean Absolute Error (MAE) and Root Mean Squared Error (RMSE) [15] as follows.

(10) $$\mathrm{MAE}=\frac{1}{N}\sum_{i=1}^{N}\left|y_{i}-{\hat{y}}_{i}\right|$$

(11) $$\mathrm{RMSE}=\sqrt{\frac{1}{N}\sum_{i=1}^{N}{\left(y_{i}-{\hat{y}}_{i}\right)}^{2}}$$

where $y_{i}$ is the ground-truth length of the *i*-th shrimp in pixels and ${\hat{y}}_{i}$ is the predicted length, and N is the total number of shrimps in the view. MAE provides the average magnitude of error, whereas the RMSE gives higher weight to large errors and reflects the overall deviation of predictions from the ground truth. The frames per second (FPS) metric was utilized to evaluate the inference performance of the model, measuring the processing speed of each algorithm, i.e., how many shrimp images can be processed per second.

## 3. Results

This section evaluates various instance and semantic segmentation models to identify the best combination for measuring shrimp size. The most effective models from each category were selected based on quantitative performance metrics and integrated into the final pipeline. The proposed approach was compared with existing approaches in the literature for centerline extraction and size estimation. Performance comparison was performed using the key metrics such as Average Precision (AP), Mean Intersection over Union (mIoU), F1-score, number of trainable parameters, size estimation error, and FPS to demonstrate the accuracy and reliability of our approach.

### 3.1. Instance Segmentation Module

The instance segmentation module is designed to accurately detect and segment individual shrimp in the input images. Several instance segmentation architectures were evaluated, and the most effective one was selected based on key performance metrics, including Average Precision (AP), number of parameters, and FPS. The model that performs best across these metrics is used to extract accurate shrimp masks. The output masks are then fed to the second module, the centerline predictive module, which estimates the centerline of a shrimp.

#### 3.1.1. Implementation Details

To develop an accurate and efficient instance segmentation system for shrimp segmentation, the shrimp dataset developed in this study was employed. This dataset includes detailed annotations for each shrimp instance in the form of both masks and bounding boxes. Implementation and evaluation were carried out on six state-of-the-art, real-time instance segmentation models: SOLOv2 [20], YOLACT [21], CondInst [22], SparseInst [23], YOLOv8 [25], and RTMDet [19]. All models are anchor-free, except for YOLACT, which uses anchor-based mechanisms. Anchor-free models are typically faster and more efficient [26], making them ideal for real-time applications due to their simpler design and lower computational overhead. To ensure consistency across experiments, ResNet-50 was employed as the backbone for all models except YOLOv8 and RTMDet, which utilize CSPDarknet [27] and CSPNeXt [24] backbones. These two models are architecturally designed around CSP-based backbones, and using their native configurations ensures optimal compatibility and performance. Each algorithm was trained for 70 epochs using a Stochastic Gradient Descent (SGD) optimizer. The learning rate was set to 0.01 with a momentum of 0.9 and a weight decay of 0.0001. A multi-step learning rate scheduler was employed to improve training stability. The experiments were conducted on NVIDIA TITAN RTX GPUs with 24 GB VRAM.

The model performance was evaluated using COCO-style metrics including Average Precision (AP) at IoU thresholds of 0.50 ${\mathrm{AP}}_{50}$, 0.75 ${\mathrm{AP}}_{75}$, and averaged AP from 0.50 to 0.95 ${\mathrm{AP}}_{50–95}$. The number of parameters and frames per second (FPS) metrics for all models are also provided. These factors help us compare the model’s size and its ability to work in real time, which are important considerations for future deployment on embedded systems.

#### 3.1.2. Experimental Results

This section presents the evaluation of instance segmentation models on the shrimp dataset. The assessment highlights the trade-offs between accuracy, model size, and real-time performance. Table 1 presents the quantitative evaluation of models on the test dataset across three distinct environments. RTMDet-m achieves the highest performance across all evaluation metrics. It achieves the highest accuracy with an ${\mathrm{AP}}_{50}$ of 0.960, ${\mathrm{AP}}_{75}$ of 0.795, and ${\mathrm{AP}}_{50–95}$ of 0.631. Additionally, it is the fastest among the models, running at 58 FPS. This strong performance and low parameter count of 34.2 million make it a great choice for real-time applications that need both accuracy and speed. YOLOv8-m also delivers strong results, attaining an ${\mathrm{AP}}_{50–95}$ of 0.630 and the highest speed among all models except RTMDet-m, at 55 FPS. CondInst achieves a competitive ${\mathrm{AP}}_{50}$ of 0.934, but its performance drops at higher IoU thresholds with an ${\mathrm{AP}}_{75}$ of 0.663 and ${\mathrm{AP}}_{50–95}$ of 0.574. It has a moderate number of parameters and a decent FPS. SOLOv2 performs reasonably well with an ${\mathrm{AP}}_{50–95}$ of 0.593 and a solid FPS of 44, although it has the highest number of parameters 46.2 M, potentially limiting its efficiency on edge devices. SparseInst and YOLACT show comparatively lower performance, particularly at stricter IoU thresholds. SparseInst, despite having a decent ${\mathrm{AP}}_{50}$ of 0.836, suffers from a low ${\mathrm{AP}}_{75}$ of 0.428. YOLACT, although efficient, yields the lowest accuracy overall, indicating limitations in mask quality and fine-grained localization.

**Figure 5:**
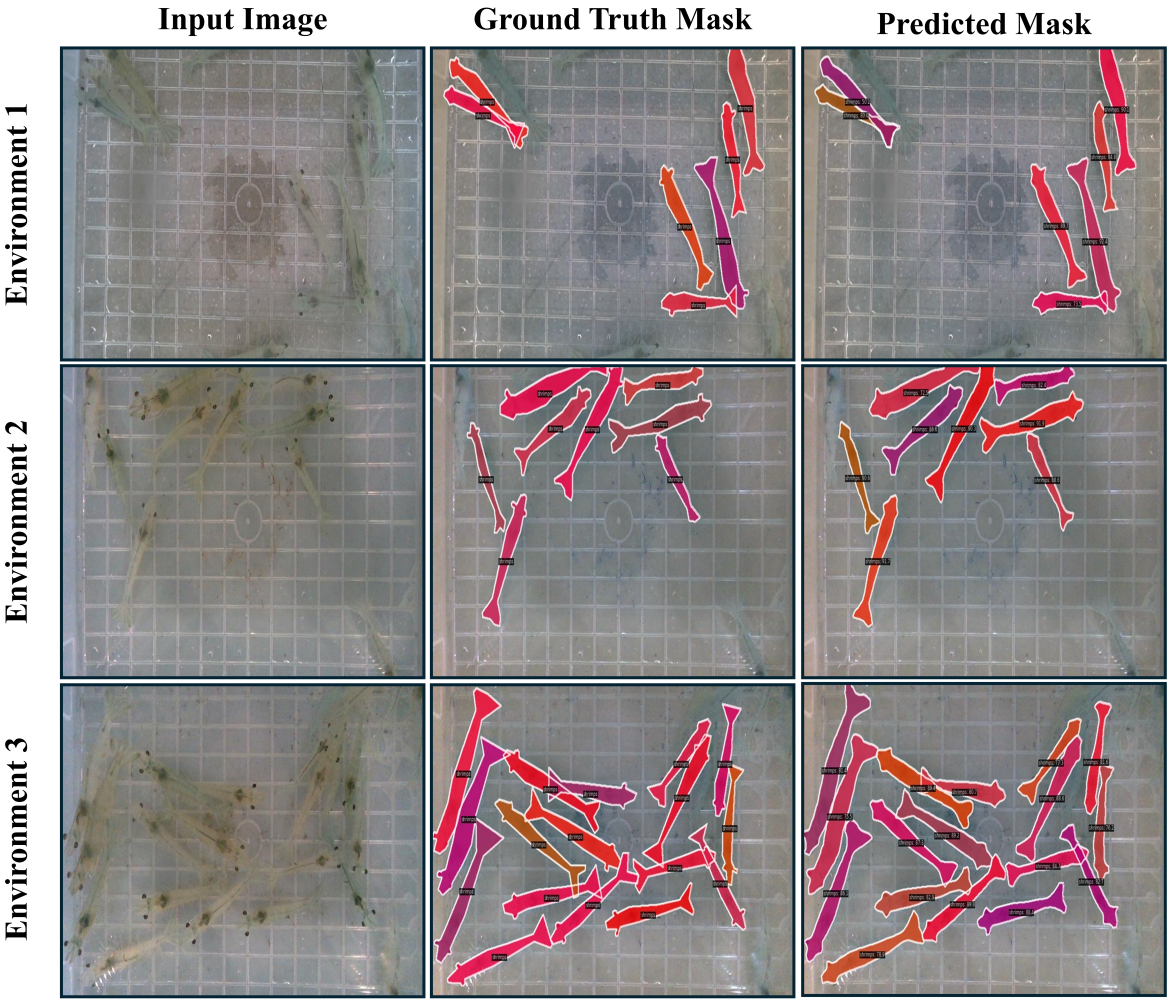
Visualization of Instance segmentation results on test data with three different environments using RTMDet-m model.

In summary, the RTMDet-m model stands out as the most effective option for both segmentation quality and real-time performance, which is closely followed by the YOLOv8-m. These findings show that the RTMDet-m method is instrumental for real-time instance segmentation of shrimps. The model demonstrates strong performance in terms of segmentation accuracy, particularly in handling challenges such as varying shrimp poses and the transparency of shrimp bodies that often blend into the background. Based on these evaluation metrics, RTMDet-m was selected as the primary model for the instance segmentation of shrimp. Figure 5 further illustrates the qualitative results of the RTMDet-m model, showcasing its impressive precision in segmenting individual shrimps under diverse conditions within our dataset.

### 3.2. Centerline Predictive Module

In the first stage of our shrimp centerline prediction, an instance segmentation model was used to detect shrimp instances and generate their individual masks. These masks are then sent to the second module, i.e., the centerline prediction module, which estimates the centerline structure using our custom segmentation algorithm. The proposed custom segmentation model shown in Figure 4 was evaluated against state-of-the-art semantic segmentation algorithms. The comparative analysis based on metrics precision, recall, Intersection over Union (IoU), F1-score, the number of trainable parameters, model size, and frames per second (FPS) demonstrates the overall superior performance of our proposed segmentation model.

#### 3.2.1. Implementation Details

A specialized dataset was constructed to predict the centerline of each individual shrimp by mapping shrimp masks to their corresponding centerlines. Each mask was manually annotated to represent a centerline, enabling the semantic segmentation algorithms to learn a pixel-wise mapping from the fused shrimp mask and RGB image to its centerline representation. Four advanced semantic segmentation models: U-Net [28], U-Net++ [29], LinkNet [30], and DeepLabv3 [31] were implemented and evaluated in comparison with the proposed model. To ensure lightweight and real-time performance, all models utilized MobileNetV2 [32] as the encoder backbone. To train the models, a Stochastic Gradient Descent (SGD) optimizer was used due to its effectiveness in navigating complex loss landscapes. The models were trained for 40 epochs, starting with an initial learning rate of 0.02. To stabilize and improve training performance, a multi-step learning rate scheduler was employed, which gradually decreases the learning rate as training progresses. All experiments and training were performed using NVIDIA TITAN RTX GPUs each with 24 GB of VRAM. The performance of each model was evaluated using several key metrics: Intersection over Union (IoU), F1-score, precision, recall, the number of trainable parameters, and FPS during inference. These metrics are useful to evaluate the accuracy and the model’s ability to perform in real time.

#### 3.2.2. Experimental Results

To evaluate the performance of semantic segmentation models for the centerline prediction, a comprehensive comparison of the segmentation models was conducted. Those models include UNet, UNet++, LinkNet, DeepLabv3 and our proposed segmentation model. To make a fair comparison, all models have the same backbone (MobileNetv2). The models were evaluated based on standard segmentation metrics, which include precision, recall, F1-score, mean Intersection over Union (mIoU), number of parameters and FPS. These metrics are essential for evaluating the accuracy and efficiency of the models. The results of all models are summarized in Table 2. Among all the models, our proposed segmentation model achieved the best overall performance across all evaluation metrics, recording the highest F1-score (0.883) and mIoU (0.791). This indicates its superior capability in accurately predicting the centerline regions. It also had the highest recall (0.900), which is a crucial strength that ensures accurate centerline detection, as missing parts of a centerline can be more problematic than slight over-segmentation. UNet++ followed closely, achieving an F1-score of 0.868 and mIoU of 0.784. Its enhanced performance over the traditional UNet can be attributed to its nested skip connections. However, this improvement comes with increased model complexity and reduced FPS. LinkNet demonstrated a strong balance between accuracy and efficiency. Although its F1-score (0.844) and mIoU (0.731) were slightly lower than those of UNet++, it required significantly fewer parameters (2.1 M) and operated faster, making it suitable for lightweight deployment scenarios. The baseline UNet performed reasonably well, achieving an F1-score of 0.843 and mIoU of 0.729, outperforming LinkNet in precision but not in overall segmentation quality. It maintained a high FPS but had a larger model size compared to LinkNet and DeepLabv3.

Our proposed model, despite having a slightly lower FPS, shows superior segmentation performance in centerline prediction compared to the baseline DeepLabv3. Specifically, it achieves higher precision (0.900 vs. 0.882), F1-score (0.883 vs. 0.860), and mean Intersection over Union (IoU). Our proposed model identifies key features while maintaining consistent performance and efficiency. It offers the best balance of speed and accuracy, providing cutting-edge performance while using fewer parameters.

**Figure 6:**
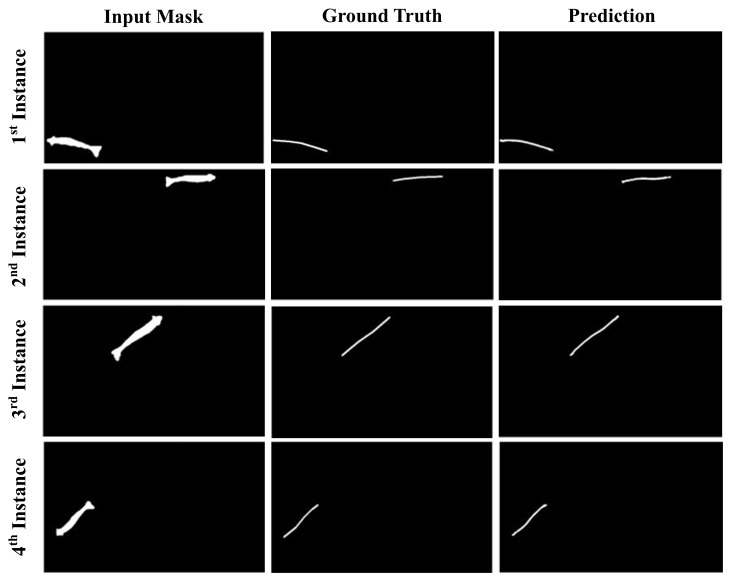
Visualization of shrimp instances, their ground truths and predicted centerlines generated using our proposed segmentation model.

Figure 6 shows centerline predictions for multiple shrimp instances from different models. Each shrimp’s centerline is accurately segmented, demonstrating the model’s ability to capture structural details and extract precise centerlines, even in complex scenes with overlapping shrimp.

### 3.3. Extraction of Centerline Skeleton for Precise Shrimp Size Estimation

The final stage of our pipeline involves the precise estimation of shrimp size based on the one-pixel-wide centerline. To begin with, the output centerline from the semantic segmentation module, which is typically a few pixels wide, is post-processed using the Zhang–Suen thinning algorithm. This algorithm systematically removes redundant edge pixels while preserving the topological structure of the centerline. A one-pixel-wide path represents the medial axis of the shrimp body. This refined line serves as a robust and scale-invariant factor for the precise measurement.

**Figure 7:**
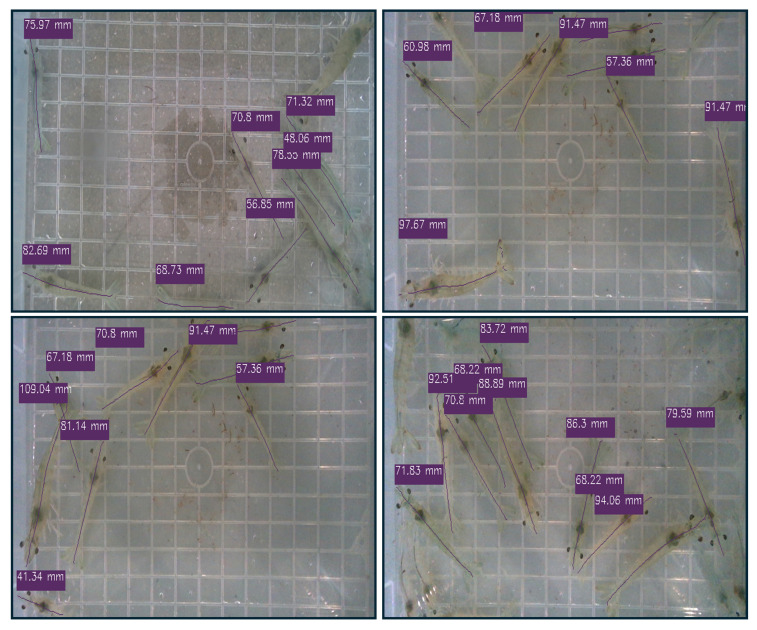
Real-time inference results of our proposed framework, displaying shrimp instances with predicted centerlines and estimated size in millimeters (mm).

This pixel length is then converted to real-world units (millimeters) using a fixed pixel-to-length ratio obtained through calibration using a reference object of known size. Our proposed framework provides the precise size estimation of individual shrimp across various environments. Figure 7 shows the results of our predictions with centerlines marked on each shrimp along with the estimated size in millimeters.

Quantitative results (Table 3) show that the proposed framework substantially outperforms the other approaches, achieving the lowest MAE (10.23 mm) and RMSE (12.79 mm), indicating highly precise size estimation. In contrast, PCFM and VDBM exhibited significantly higher errors, while U-Net with SAG kernels provided moderate improvements but still lagged behind the proposed framework.

Furthermore, the proposed framework maintains 5.14 FPS, demonstrating a favorable trade-off between accuracy and computational efficiency. The qualitative results shown in Figure 7 further confirm the robustness of the approach, illustrating more accurate and consistent shrimp centerline predictions. Collectively, these results validate the effectiveness of the proposed framework in predicting shrimp centerlines and accurately estimating shrimp size.

### 3.4. Comparative Study of Methods for Measuring Shrimp Size

In this section, the comparison of our dual-segmentation framework for estimating shrimp size with several existing methods in the literature is presented. The proposed framework combines RTMDet-based instance segmentation with our custom segmentation model to facilitate precise shrimp centerline prediction. This is followed by a skeletonization step that transforms the centerline into a one-pixel representation to calculate the size in millimeters. Through extensive experiments in our study, it is found that the integration of the RTMDet model and our custom centerline segmentation model provides excellent results. This combination offers both high accuracy and FPS. This makes our framework well suited for deployment in practical aquaculture settings.

To assess the effectiveness of our proposed framework, our study provides both quantitative and qualitative comparisons with three notable approaches from the literature for centerline extraction. These approaches include methods such as polynomial curve fitting, the Voronoi diagram-based method, and the deep learning-based silhouette segmentation method. The detailed comparison of those methods is discussed in the following paragraph.

The method proposed by Kim et al. [13] uses polynomial curve fitting to estimate the centerline of rainbow trout. This approach allows for a precise geometric interpretation of the centerline. However, it heavily depends on preprocessing steps, especially the horizontal alignment of the object’s outline. This alignment is accomplished by estimating the principal direction using image moments. However, it is essential to note that this technique is sensitive to posture and orientation variations commonly encountered in natural aquatic environments. Additionally, the method requires manual or heuristic selection of the polynomial degree, which can be quite challenging, especially in cases where shrimp postures overlap or are non-linearly distorted. These complexities make the polynomial fitting approach less generalizable and difficult to automate across various datasets.

Zhao et al. [33] proposed a technique based on Voronoi diagrams to extract the medial axis of objects. This method was adapted for estimating the centerlines of shrimp silhouettes. While it effectively captures the overall structure of the shape, it tends to introduce spurious branches, commonly referred to as “burrs,” particularly in the head and tail regions. These artifacts distort size measurements and necessitate additional pruning steps. To address this issue, a graph-based algorithm was implemented to retain the longest path as the centerline. However, the additional computational burden of this post-processing significantly reduces throughput, which hinders real-time applicability and increases system complexity.

Wang et al. [3] present a deep learning-based approach that combines U-shaped fully convolutional networks (U-Net) with SAG kernels to estimate the centerline from Artemia silhouettes. While this method is effective at modeling complex morphological features, it is limited by the challenges associated with silhouette-based segmentation. In multi-object scenarios, overlapping individuals are merged into a single foreground region, preventing the separation of instances. Additionally, the approach struggles with different orientations of shrimp, often leading to incomplete or noisy segmentation masks. This results in inaccurate or disconnected centerlines, making the instance-level identity hard, which is essential for individual size measurement.

**Figure 8:**
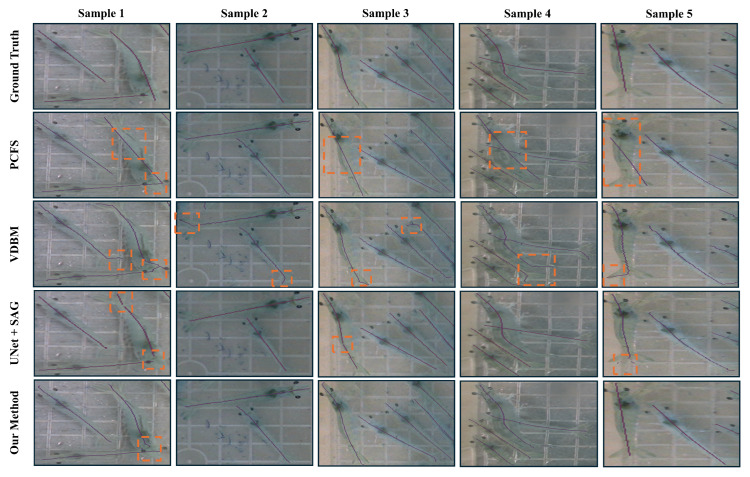
Qualitative results of convential and deep learning based methods for finding the centerline of the shrimp. The orange dashed boxes indicates regions where centerline predictions mismatch from the ground-truth centerlines.

Among the evaluated methods, the polynomial curve-fitting method (PCFM) provides the highest FPS due to its simplicity and the absence of learning-based components. Its speed advantage comes from being a conventional, model-free approach. However, thePCFM faces difficulty in accurately estimating centerlines when shrimp shapes become complex or irregular. As shown in Figure 8 (samples 1 and 5), the fitted curves deviate from the true centerline, resulting in misleading outputs. Additionally, the PCFM requires parameter tuning, which is impractical for shrimp because of their inconsistent morphology. The Voronoi diagram-based method produces reasonably accurate centerlines, particularly in the mid-body regions. However, toward the tail, the method often selects the longest path without considering anatomical accuracy due to its inherent algorithmic structure. Additionally, this method tends to generate many extraneous branches, or “burrs,” which must be removed using a graph-based approach to isolate the longest valid path. While this preprocessing step is somewhat effective, it introduces significant computational overhead, resulting in a notably lower FPS.

The UNet+SAG performs well, mainly when supported by high-quality instance masks. It provides robust centerline predictions, though its FPS is moderate. In contrast to the above-mentioned approaches, our proposed framework strikes the best balance between accuracy and efficiency. It consistently outperforms all baseline methods in terms of Mean Absolute Error and Root Mean Squared Error (MSE) while providing a reasonable FPS.

To evaluate shrimp size estimation methods, both pixel-based measurements and real-world dimensions were used. Our dataset includes ground-truth lengths in pixel units and millimeters, allowing us to assess the model’s accuracy in both domains for practical aquaculture applications. The primary metrics used in our comparative study are Mean Absolute Error (MAE), which measures the average deviation between predicted and actual lengths, and Root Mean Squared Error (RMSE), which highlights larger errors. These metrics are reported in both pixels and millimeters in Table 3 for quantitative comparison.

## 4. Discussion

This work presents a dual-segmentation framework for centerline prediction in shrimp and achieves the highest segmentation accuracy and processing speed in terms of FPS. The system is designed to support real-time processing, which is crucial for measuring shrimp in aquaculture environments. The following discussion will provide insights into quantitative results from each stage of our framework along with a comparative evaluation against state-of-the-art methods.

During the evaluation phase, first-instance segmentation models were compared that provide instance segmentation results for shrimp. Among the models tested, RTMDet-m demonstrated the most consistent and superior performance. It achieved an average precision (AP_50_) of 0.960, outperforming other models such as SOLOv2 (AP_50_ = 0.899), YOLACT (AP_50_ = 0.902), CondInst (AP_50_ = 0.934), SparseInst (AP_50_ = 0.836) and YOLOv8-m (AP_50_ = 0.931). In addition to this high precision, RTMDet-m attained an AP_75_ of 0.795 and a COCO-style mean average precision (AP_50–95_) of 0.631, indicating its high performance in instance segmentation of shrimp. The model’s ability to accurately segment instances at higher IoU thresholds shows that the model can create accurate masks around the complex shapes of shrimp, which is crucial for precise measurements. RTMDet-m achieved this level of accuracy while running at 58 FPS, outperforming other baseline models.

The second part of the evaluation provides a comparison between our proposed centerline segmentation model and other baseline methods. This task is particularly challenging due to the nature of shrimp bodies and their varying postures. To address this challenging task, our study proposed a custom segmentation model followed by a morphological skeletonization (MS) process for shrimp centerline prediction. The combination of our proposed segmentation model and MS proved to be the most effective among other baseline methods. It achieved the highest F1-score of 0.883 and a mean Intersection-over-Union (mIoU) of 0.791. These results indicate both high pixel-wise accuracy and strong spatial alignment with the ground-truth centerline. The method achieved a highest FPS of 150, which is essential for real-time applications. While other models like UNet++, LinkNet, and DeepLabv3 had competitive F1-scores of 0.868, 0.844, and 0.860, respectively, they showed lower FPS and produced slightly lower segmentation quality. The superior performance of our proposed segmentation model for centerline prediction can be attributed to the Atrous Spatial Pyramid Pooling (ASPP) and a UNet-type feature fusion mechanism, which effectively captures multi-scale context.

To validate the effectiveness of our framework, the accuracy of shrimp centerline prediction and its size are compared with existing methods reported in the above paragraphs. Our analysis revealed that our framework achieved the lowest mean absolute error (MAE) of 1.02 centimeters (cms) compared to other baseline methods. This marks a significant upper hand over competing methods, such as PCFM, which recorded an MAE of 4.46 cms, and VDBM with an MAE of 5.07 cms. The UNet+SAG deep learning based approach also reported a close but still high MAE of 3.02 cms. Additionally, our framework provides a higher FPS of 5.14 compared to other methods for centerline prediction.

In summary, our dual-segmentation framework provides accurate results for instance segmentation and centerline prediction, outperforming state-of-the-art methods in terms of accuracy metrics and operational speed. The integration of RTMDet-m and our improved segmentation model shows the effectiveness of our proposed framework for shrimp size estimation by achieving the lowest MAE and RMSE.

## 5. Conclusions

This study presents a robust dual-segmentation framework for the size measurement of shrimp using center-line predictions, providing an accurate and non-intrusive length estimation method. The system is designed to precisely predict the individual shrimp size regardless of their shape or posture. In the first stage, the RTMDet-m model was employed for high-speed instance segmentation, achieving a high average precision (AP_50_) of 96%. This performance surpasses that of other instance segmentation models in both accuracy and FPS. In the second stage, the centerline of individual shrimp was extracted using our proposed custom segmentation model. The proposed centerline segmentation model in this study demonstrated exceptional performance with an F1-score of 88.3%. Our proposed framework outperforms other conventional and deep learning based methods, providing the lowest mean absolute error of 1.02 cm. This framework represents a significant advancement in aquaculture management, allowing for continuous and stress-free monitoring of shrimp growth. Additionally, it can be adapted for other aquatic species, such as fish, enabling early disease detection and overall monitoring of stock health, all without disrupting the natural behavior.

## Figures and Tables

**Table 1:** Performance comparison of RTMDet-m model with other real-time instance segmentation models in terms of accuracy, number of parameters, and FPS.

Model	${\mathrm{AP}}_{50}$	${\mathrm{AP}}_{75}$	${\mathrm{AP}}_{50–95}$	Params (M)	FPS
SOLOv2	0.899	0.724	0.593	46.2	44
YOLACT	0.783	0.461	0.437	34.7	43
CondInst	0.934	0.663	0.574	37.5	42
SparseInst	0.836	0.428	0.432	42.6	43
YOLOv8-m	0.931	0.735	0.630	27.2	55
RTMDet-m	**0.960**	**0.795**	**0.631**	**34.2**	**58**

Bold values indicate the best result for each metric among the compared models.

**Table 2:** Quantitative comparison of semantic segmentation models with our proposed model for shrimp centerline prediction.

Model	Precision	Recall	F1-Score	mIoU	Param (M)	FPS
UNet	0.839	0.847	0.843	0.729	4.4	164
UNet++	0.864	0.893	0.868	0.784	4.6	127
LinkNet	0.826	0.863	0.844	0.731	2.1	160
DeepLabV3	0.842	0.882	0.860	0.754	2.1	**169**
Our Proposed Model	**0.866**	**0.900**	**0.883**	**0.791**	**2.1**	**150**

Bold values indicate the best result for each metric among the compared models.

**Table 3:** Quantitative comparison of centerline extraction methods with our proposed framework using MAE and RMSE for size estimation in both pixels and real size along with FPS for evaluating real-time performance.

Model	MAE (px)	MAE (mm)	RMSE (px)	RMSE (mm)	FPS
PCFM	84.76	44.61	105.95	55.76	10
VDBM	96.27	50.67	120.34	63.34	0.05
UNet+SAG	57.40	30.21	71.75	37.21	3.2
Our Framework	**19.44**	**10.23**	**24.30**	**12.79**	**5.14**

Bold values indicate the best result for each metric among the compared methods.

## Data Availability

The data presented in this study are available upon request from the corresponding author.
